# Genetic Transformation of *Torenia fournieri* L. with the *Bacillus thuringiensis Cry1Ab* Gene Confers Resistance to *Mythimna separata* (Walker)

**DOI:** 10.3390/plants13243568

**Published:** 2024-12-20

**Authors:** Lin Chen, Pei Wang, Lixia Tan, Houhua Li, Dun Wang

**Affiliations:** 1 College of Plant Protection, Yangzhou University, Yangzhou 225009, China; chenlin88@yzu.edu.cn; 2 State Key Laboratory of Crop Stress Biology for Arid Areas, College of Plant Protection, Northwest A&F University, Yangling, Xianyang 712100, China; jlwp0306@163.com (P.W.); tanlixia-000@163.com (L.T.); 3 Qingdao Smart Village Development Service Center, Qingdao 266000, China; 4 College of Landscape Architecture and Art, Northwest A&F University, Yangling, Xianyang 712100, China; lihouhua@nwafu.edu.cn

**Keywords:** transgenic Torenia, *Cry1Ab*, insect resistance, oriental armyworm, protective enzymes

## Abstract

*Torenia fournieri* L. is a popular ornamental plant in the genus Torenia, widely used in commercial landscaping, especially during the summer. Additionally, Torenia has served as a model ornamental plant in many studies exploring ornamental characteristics and pest control through genetic engineering. To date, no research has been reported on developing insect-resistant Torenia expressing genes from *Bacillus thuringiensis* (Bt). In this study, a recombinant vector carrying the *Cry1Ab* gene from Bt, pBI121-*Cry1Ab*, was constructed and transferred into *T. fournieri* via *Agrobacterium tumefaciens*-mediated transformation. A total of 13 shoots survived on the kanamycin selection medium, among which four putative transgenic lines, designated L1, L2, L7, and L11, were molecularly confirmed by PCR and Southern blot analysis, indicating successful integration of the *Cry1Ab* gene into the genomes of these lines. Quantitative real-time PCR and ELISA results further verified the successful expression of the *Cry1Ab* gene in the leaves of all four transgenic lines. Insect bioassay results demonstrated that all four transgenic lines showed strong resistance to the insect pest, *Mythimna separata*, with mortality rates ranging from 59.9% to 100.0%, in contrast to a larval mortality rate of 16.2% in the wild-type Torenia. Additionally, these transgenic lines significantly decreased in larval survival rates compared to those fed on wild-type plants. Furthermore, these transgenic lines activated superoxide dismutase (SOD) activity at 12 and 24 h, and catalase (CAT) activity at 72 h, while suppressing SOD activity at 72 h, and peroxidase (POD) activity over time. Our findings indicate that these transgenic lines exhibit high resistance to the insect pest and provide new insights into controlling insect pests in ornamental plants through genetic approaches.

## 1. Introduction

Insect pests are among the most harmful factors that cause billions of dollars of crop losses and pose ongoing challenges for growers worldwide [1,2]. Although chemical pesticides have provided considerable protection for crops against insect pests, the widespread and indiscriminate use of these chemical insecticides has resulted in environmental pollution, adverse effects on non-target organisms, and increasing health risks for humans and animals due to pesticide residues in food products [3,4,5]. Therefore, the development and adoption of insect-resistant cultivars have been considered as the most cost-effective and environmentally friendly strategy for pest management [6,7]. However, developing insect-resistant crop varieties through conventional plant breeding procedures is both time-consuming and challenging, owing to the complexity of quantitative traits governed by multiple loci, achieving only limited success to date [8,9]. As an alternative, introducing insecticidal toxin genes through biotechnological approaches, such as *Agrobacterium*-mediated genetic transformation, offers a promising solution for developing novel plant resistance traits that provide effective and durable protection against a broad spectrum of insect pests.

In the past decades, a number of novel candidate genes from various sources, such as microbes, plants, and animals, encoding insecticidal proteins like *Bacillus thuringiensis* (Bt) toxins (e.g., Cry proteins) [10,11], plant lectins [12], and protease inhibitors [13,14], have been identified and introduced into crops to develop insect-resistant varieties [15]. Among them, *B. thuringiensis* has emerged as one of the most significant sources of insect-resistant genes [16]. *B. thuringiensis* is a gram-positive, spore-forming bacterium that produces insecticidal proteinaceous crystals (commonly known as crystal proteins or Cry toxins) during sporulation. These crystals are activated by insect gut proteases and subsequently bind to specific protein receptors in the midgut, leading to the death of the target insect pests [17,18,19]. Various *Cry* genes (or modified *Cry* genes), including *Cry1Ab*, *Cry1Ac,* and *Cry2Ab,* originally derived from *B. thuringiensis*, have been widely used to develop genetically modified crops that confer insect resistance [10,16]. To date, transgenic crops expressing *Bt* genes have been extensively cultivated commercially, including Bt corn expressing *Cry1Ab* [20], Bt cotton expressing *Cry1Ac* and *Cry2Ab* [21], and Bt soybean expressing *Cry1Ac* [22]. However, less attention has been given to ornamental plants in expressing *Bt* genes for resistance against insect pests.

*Torenia fournieri* L., commonly known as Torenia or wishbone flower, is one of the most important species in the genus *Torenia* for commercial ornamental use, especially during the summer [23,24]. This popular annual plant is widely appreciated for its diverse floral colors, including blue, white, pink, and violet [23,24]. Additionally, Torenia has a small genome size similar to that of Arabidopsis, a short lifespan [25], and benefits from the establishment of a simple and efficient *Agrobacterium*-mediated genetic transformation system [26,27], as well as the application of CRISPR/Cas9 genome editing technology [28]. These features make Torenia a horticultural model plant for many genetic engineering studies of floral traits and for exploring gene functions that regulate flower organ development [23,26,27,29]. For example, ectopically expressing three betalain-biosynthetic genes, namely *BvCYP76AD1* from *Beta vulgaris, MjDOD* (dihydroxyphenylalanine 4,5-dioxygenase), and *MjcDOPA5GT* (cyclo-DOPA-5-O-glucosyltransferase) from *Mirabilis jalapa*, in the purple-flowered Torenia cultivar Crown Violet induced the accumulation of two betacyanins, betanin and isobetanin, leading to a modification of the flower color from purple to reddish [30]. The roles of *TfALOG3* and *TfBOP2*, which are specifically expressed in the corolla neck and proximal corolla regions, respectively, in the proximodistal differentiation of the corolla, were analyzed through the generation of loss-of-function mutants; furthermore, TfALOG3 can recruit TfBOP2 to the nuclear region, forming a BOP-ALOG complex that regulates petal proximodistal differentiation in Torenia [31,32].

Recently, several reports have focused on disease and pest control using Torenia as the experimental plant. For instance, transgenic Torenia plants overexpressing Arabidopsis agmatine coumaroyltransferase (AtACT) gene, which catalyzes the last step in the biosynthesis of antifungal hydroxycinnamic acid amides (HCAAs), exhibited enhanced resistant to the necrotrophic fungus *Botrytis cinerea* [33]. Shimoda et al. [34] found that *T. fournieri* plants infested by the two-spotted spider mite *Tetranychus urticae* scarcely release herbivore-induced plant volatiles (HIPVs), whereas *T. hybrida* released a complex blend of HIPVs, which were attractive to the predatory mite *Phytoseiulus persimilis*. To date, no study has been reported on transgenic Torenia expressing insecticidal genes to improve resistance against insects. Additionally, the potential of transgenic Torenia plants as an ideal platform for pest control in ornamental plants has not yet been assessed.

Ornamental plants attacked by lepidopteran insects in field conditions can lead to significant losses, as even minor damage can substantially diminish their ornamental value [29,35]. Thus, using torenia as a model to evaluate the potential of expressing toxic protein-encoding genes may provide a basis for pest control in ornamental plants through genetic engineering approaches. The oriental armyworm, *Mythimna separata* (Walker), is a devastating insect pest that feeds on a wide variety of host plants, including vegetables, cereals, fruits, and ornamental plants [36,37]. Its seasonal long-distance migration, high reproductive potential, and widespread distribution make it a significant challenge to agricultural production [38]. Moreover, *M. separata* is commonly used in bioassays to test the resistance of *Bt* transgenic plants to insect pests [16,39]. In this study, we constructed the recombinant vector pBI121-*Cry1Ab* and transferred it into Torenia via *Agrobacterium*-mediated transformation. Four transgenic Torenia lines expressing the *Cry1Ab* gene were selected and molecularly confirmed, designated L1, L2, L7, and L11. Their insecticidal activity against the oriental armyworm, *Mythimna separata* (Walker), was evaluated. Furthermore, we assessed the effects of transgenic plants expressing the *Cry1Ab* gene on the activity of protective enzymes in *M. separata*. Our study is the first to develop transgenic Torenia plants expressing the *Cry1Ab* gene for insect resistance, providing new insights into controlling insect pests in ornamental plants through genetic approaches.

## 2. Results

### 2.1. Construction of Recombinant Vector pBI121-Cry1Ab

**Figure 1:**
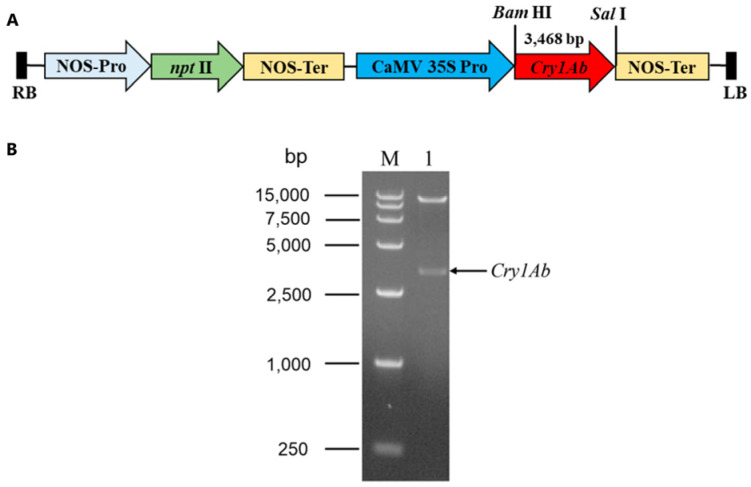
Construction of recombinant vector pBI121-*Cry1Ab* used for Torenia transformation. (**A**) Schematic diagram of the recombinant construction of pBI121-*Cry1Ab* harboring the kanamycin resistance gene *nptII* driven by the NOS promoter. RB and LB, right and left borders of T-DNA reg; NOS-Pro, nopaline synthase promoter; NOS-Ter, nopaline synthase terminator; CaMV 35S Pro, cauliflower mosaic virus 35S promoter. (**B**) The recombinant construction of pBI121-*Cry1Ab*. (**B**) Restriction analysis of recombinant plasmid pBI121-*Cry1Ab* with endonucleases *Bam*HI and *Sal*I. Lane M, DNA marker DL 15,000 (TaKaRa Biotechnology (Dalian) Co., Ltd., Dalian, China); lane 1, digested pBI121-*Cry1Ab* plasmid.

In order to obtain the *Cry1Ab*-transgenic Torenia plants, we cloned the full-length coding sequence (CDS) of the *Cry1Ab* gene (GenBank accession number AF358861.1) from the *B. thuringiensis* strain C3. The full-length CDS of the *Cry1Ab* gene, 3468 bp in size, was subsequently sub-cloned into the plant binary vector pBI121 using *Bam*HI and *Sal* I to yield the recombinant vector pBI121-*Cry1Ab* (Figure 1A). The *Cry1Ab* gene was driven by the CaMV 35S promoter. The recombinant vector pBI121-*Cry1Ab* was confirmed through a restriction analysis using the endonucleases *Bam*HI and *Sal*I. Upon restriction, two fragments of different lengths were released (Figure 1B). This confirmed that plasmid pBI121-*Cry1Ab* was transferred into the *A. tumefaciens* strain LBA4404 for subsequent Torenia transformation.

### 2.2. Development of Transgenic Torenia Plants

**Figure 2:**
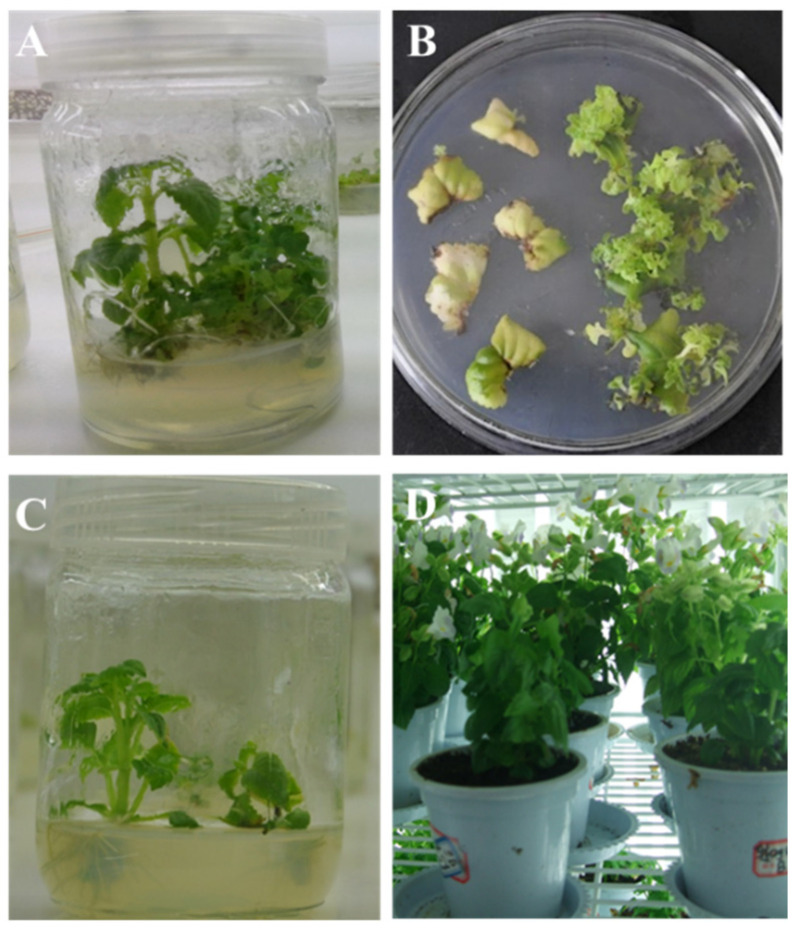
The generation of *Cry1Ab*-transgenic Torenia plants. (**A**) The 4-week-old in vitro Torenia plants; (**B**) *Agrobacterium*-infected leaf discs on the selection medium; (**C**) elongation of kanamycin-resistant shoots on the shoot elongation medium; (**D**) putative transgenic Torenia plants in a greenhouse chamber.

To generate transgenic Torenia plants expressing *Cry1Ab* gene, the recombinant plasmid pBI121-*Cry1Ab* was transformed into Torenia by *Agrobacterium*-mediated transformation. The leaf discs from 4-week-old in vitro Torenia plants (Figure 2A) were cut into 0.25 cm^2^ pieces and were co-cultivated with the *Agrobacterium* suspension as described in the Materials and Methods section. After 4-6 weeks of culturing *Agrobacterium*-infected leaf discs on the selection medium, small adventitious buds sprouted from the calli, while the edges of the untransformed leaves turned brown (Figure 2B). A total of thirteen shoots survived on the kanamycin selection medium and were selected for further elongation and regeneration (Figure 2C). Within three months after *Agrobacterium* infection, these thirteen putative transgenic plantlets were transferred to soil and maintained in a controlled greenhouse chamber (Figure 2D).

### 2.3. Molecular Confirmation of the Transgenic Plants

**Figure 3:**
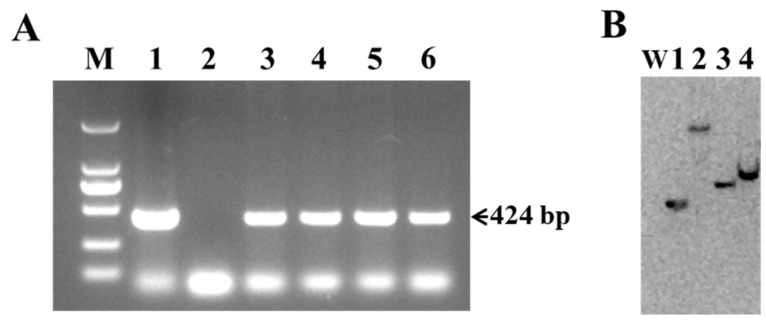
Molecular analysis of *Cry1Ab*-transgenic Torenia lines. (**A**) PCR-amplified fragments of putative transgenic lines and wild-type plants using *Cry1Ab*-specific primers. Lane M, DL2000 marker (TaKaRa Biotechnology (Dalian) Co., Ltd.); lane 1, plasmid pBI121-*Cry1Ab* (positive control); lane 2, wild-type Torenia (negative control); lane 3-6, transgenic lines L1, L2, L7, and L11. (**B**) Southern blot analysis of *Hin*dIII-digested genomic DNA isolated from PCR-positive transgenic lines. W, wild-type Torenia; lanes 1-4, transgenic lines L1, L2, L7, and L11.

Genomic DNA isolated from the leaves of putative transgenic lines was used to confirm the presence of the *Cry1Ab* gene through PCR and Southern blot analysis. As shown in Figure 3A, four of the thirteen putative transgenic lines, designated L1, L2, L7, and L11, detected the expected 424 bp fragment of *Cry1Ab*, along with the positive control (pBI121-*Cry1Ab* plasmid). No amplification was observed in the wild-type Torenia, which was used as a negative control (Figure 3A). Furthermore, the integration and copy number of the *Cry1Ab* gene in the genome of transgenic Torenia lines were analyzed using Southern blot. Genomic DNA isolated from four PCR-positive transgenic lines was digested with *Hin*dIII and hybridized with DIG-labeled probes. A single band was detected in all four transgenic lines, indicating the integration of the *Cry1Ab* gene in the genome of transgenic Torenia lines, while no band was observed in the genomic DNA sample from wild-type Torenia (Figure 3B).

### 2.4. Expression Levels of Cry1Ab Gene in the Transgenic Torenia Plants

**Figure 4:**
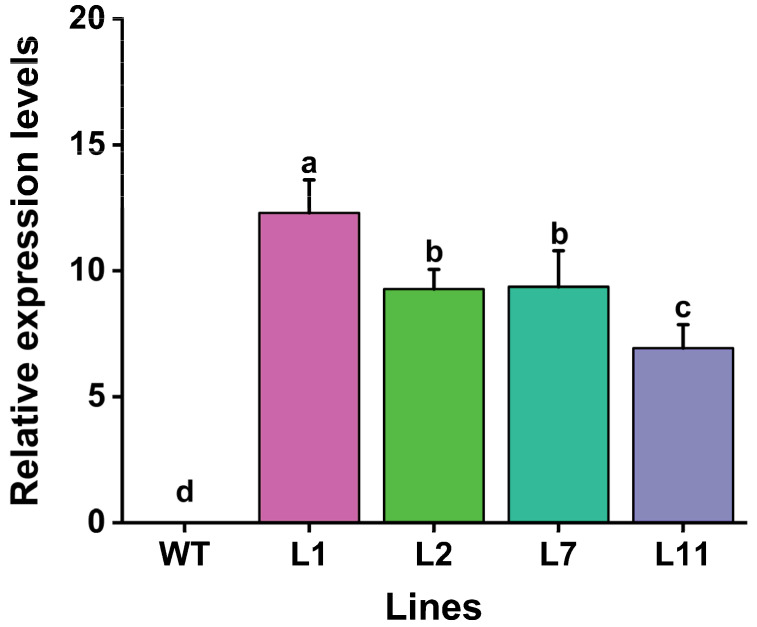
Levels of *Cry1Ab* transcripts in the leaves of four transgenic Torenia lines and wild-type plants. WT, wild-type plants; L1, L2, L7, and L11, transgenic lines expressing the *Cry1Ab* gene. Data are presented as means + standard error. The *β-Actin* gene was used as an internal standard to normalize *Cry1Ab* expression. Different lowercase letters above the bars indicate significant differences among the plant lines (one-way ANOVA followed by Tukey’s honestly significant difference (HSD) post hoc test, *p* < 0.05).

In order to determine the expression levels of the *Cry1Ab* gene in transgenic lines, quantitative real-time PCR (qRT-PCR) was performed using specific primers, as described in Table S2. The results showed that the transcripts of *Cry1Ab* were expressed in all four PCR- and Southern blot-positive lines (Figure 4). The transcription levels of the *Cry1Ab* gene in the four transgenic Torenia lines ranged from 6.9-fold to 12.3-fold, with the highest expression in line L1, followed by L7, and the lowest expression in L11. No expression was observed in the non-transformed control (Figure 4).

### 2.5. Cry1Ab Protein Abundance in Transgenic Torenia Plants

**Figure 5:**
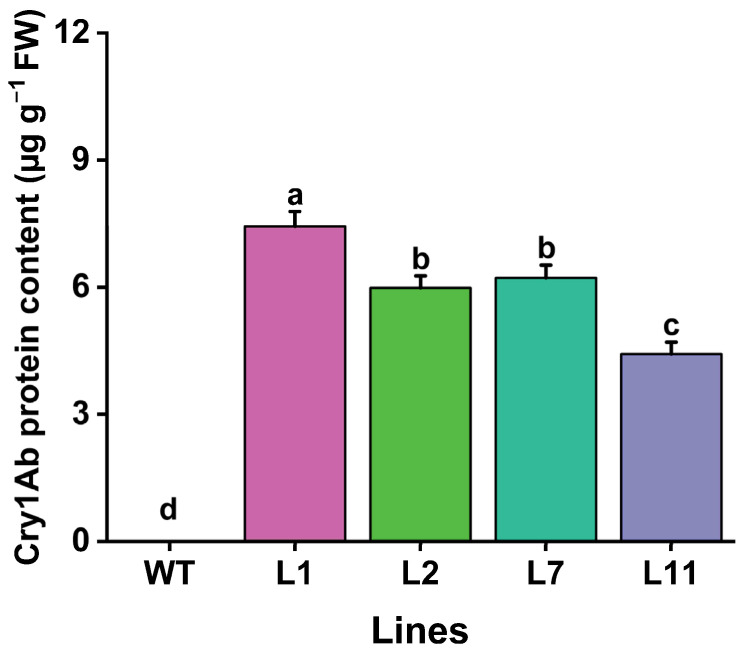
Levels of Cry1Ab protein content in the leaves of transgenic Torenia and wild-type plants. WT, wild-type plants; L1, L2, L7, and L11, transgenic lines expressing the *Cry1Ab* gene. Data are presented as means + standard error. Different lowercase letters above the bars indicate significant differences among the plant lines (one-way ANOVA followed by Tukey’s HSD post hoc test, *p* < 0.05).

The levels of Cry1Ab toxic protein in the leaves of four transgenic Torenia lines were evaluated using enzyme-linked immunosorbent assay (ELISA). Our results showed that Cry1Ab protein was detected in leaves of all four transgenic Torenia lines. The levels ranged from a maximum of 7.44 µg g^−1^ fresh weight in L1 leaves to a minimum of 4.43 µg g^−1^ fresh weight in L11. No Cry1Ab protein was detected in the leaves of wild-type plants (Figure 5).

### 2.6. Transgenic Torenia Plants Expressing the Cry1Ab Gene Are Resistant to M. separata

**Figure 6:**
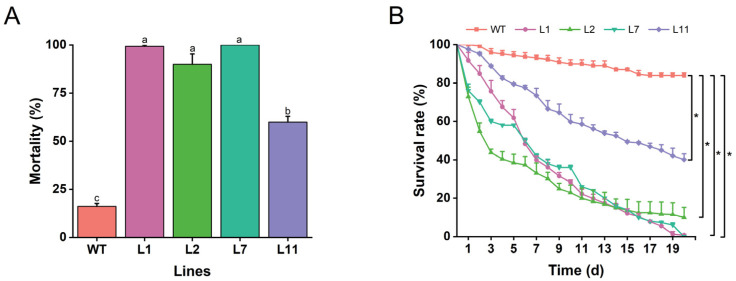
Resistance of transgenic plants expressing the *Cry1Ab* gene to *Mythimna separata*. The mortality rate (**A**) and survival rate (**B**) of *Mythimna separata* larvae fed on leaves from transgenic and wild-type plants. WT, wild-type plants; L1, L2, L7, and L11, transgenic lines expressing the *Cry1Ab* gene. Data are presented as means + standard error. Different lowercase letters above the bars and asterisks indicate significant differences between transgenic lines and wild-type plants (one-way ANOVA followed by Tukey’s HSD post hoc test, *p* < 0.05; *, *p* < 0.05).

To evaluate the effects of transgenic Torenia plants expressing the *Cry1Ab* gene against *M. separata*, all four confirmed transgenic lines, L1, L2, L7, and L11, along with wild-type plants were exposed to neonatal larvae of *M. separata*. The mortality rate of larvae fed on leaves of transgenic lines ranged from 59.9% to 100.0%, significantly higher than those fed on wild-type Torenia. Notably, transgenic lines L1, L2, and L7 exhibited high resistance against larvae, with mortality rates of 99.3%, 90.0%, and 100.0%, respectively (Figure 6A). Consistent with the mortality rates, the survival rates of *M. separata* from neonates to pupation were significantly lower on four transgenic lines compared to wild-type plants (Figure 6B). These findings indicate that the expression of *Cry1Ab* gene in Torenia effectively controls *M. separata*.

### 2.7. Transgenic Torenia Plants Expressing the Cry1Ab Gene Affect the Activities of Protective Enzymes in M. separata

**Figure 7:**
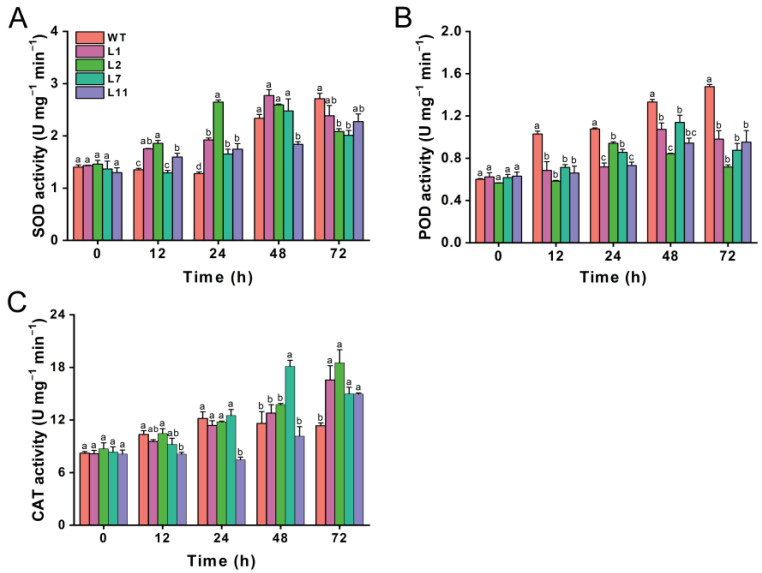
Effect of transgenic plants expressing the Cry1Ab gene on the activities of protective enzymes in *Mythimna separata.* Activities of SOD (**A**), POD (**B**), and CAT (**C**) in *Mythimna separata* larvae fed on leaves from transgenic and wild-type plants. WT, wild-type plants; L1, L2, L7, and L11, transgenic lines expressing the *Cry1Ab* gene. Data are presented as means + standard error. Different lowercase letters above bars indicate significant differences between transgenic lines and wild-type plants (one-way ANOVA followed by Tukey’s HSD post hoc test, *p* < 0.05).

We also investigated the effects of transgenic Torenia plants expressing the *Cry1Ab* gene on the activities of three important protective enzymes, including superoxide dismutase (SOD), peroxidase (POD), and catalase (CAT), in *M. separata* larvae that fed on leaves from both transgenic and wild-type plants. The SOD activity in *M. separata* larvae was higher at 12 and 24 h after feeding on transgenic lines compared to those fed on wild-type Torenia. However, SOD activity decreased at 72 h in larvae fed on the transgenic lines compared to the control plant, although line L1 and L11 did not show a significant decline relative to the control (Figure 7A). The POD activity in *M. separata* larvae fed on all four transgenic plants was significantly lower than that in those fed on wild-type Torenia at 12, 24, 48, and 72 h after release (Figure 7B). There was no difference in CAT activity at 12, 24, and 48 h, except that CAT activity in larvae fed on L11 was lower at 12 and 24 h, while CAT activity in larvae on the L7 was higher at 48 h compared to those fed on wild-type plants. However, the CAT activity in *M. separata* larvae fed on transgenic lines was obviously higher than in those fed on wild-type plants at 72 h after release (Figure 7C).

## 3. Discussion

Visible damage to plants or cut flowers caused by insect pests significantly reduces the value of ornamental plants, rendering the products unmarketable or unacceptable for export. This situation has resulted in a zero-tolerance policy for pests in commercial end products of ornamental plants, creating a strong demand for insect-resistant ornamental plants [29,35]. To date, research on transgenic insect-resistant ornamental plants through genetic engineering has been quite limited. However, transgenic ornamental plants expressing toxic protein genes from *B. thuringiensis* have been successfully developed in petunia and chrysanthemum, demonstrating high mortality rates in lepidopteran larvae [40,41,42,43]. Given the significant success in the commercial release of transgenic maize, soybean and cotton with resistance to lepidopteran pests [16,22], it appears feasible to develop transgenic ornamental plants for insect resistance using Torenia as a model.

In this study, we cloned the full CDS sequence of the *Cry1Ab* gene and constructed the binary vector pBI121-*Cry1Ab* (Figure 1), which was subsequently introduced into *T. fournieri* L. via *Agrobacterium*-mediated transformation (Figure 2). Out of thirteen lines that survived on a kanamycin selection medium, four, designated L1, L2, L7, and L11, were confirmed through PCR and Southern blot analysis, demonstrating successful integration of the exogenous *Cry1Ab* gene into the Torenia genome, achieving a transformation efficiency of 30.8% (Figure 3). Additionally, qRT-PCR and ELISA results confirmed the successful expression of the *Cry1Ab* gene in the leaves of all four transgenic lines (Figure 4 and Figure 5). Furthermore, these transgenic Torenia plants exhibited high resistance to the oriental armyworm (*M. separata*) larvae (Figure 6). Furthermore, the expression of *Cry1Ab* also affected the activity of three protective enzymes, SOD, POD, and CAT, in *M. separata* larvae (Figure 7). These findings suggest that the insertion of the exogenous *Cry1Ab* gene into Torenia offers a viable approach to developing insect-resistant plants.

An efficient regeneration and transformation method is crucial for the development of stable transgenic plants. In particular, *Agrobacterium*-mediated genetic transformation offers relatively high efficiency and stable T-DNA delivery [44], reproducibility of normal phenotypes, and stable expression of foreign genes [45], making it the primary method for transferring genes into plants. With a focus on ornamental characteristics, many genetically modified Torenia plants have been obtained via the *Agrobacterium*-mediated transformation method [26]. Reports on traits such as floral organ development [32], flower longevity [46], and modifications of flower colors and shapes [30,47,48] of Torenia demonstrate the utility of this transformation system for fundamental research. Therefore, we utilized the Torenia transformation system to explore the potential of genetically engineering transgenic ornamental plants with insecticidal traits. In this study, four transgenic lines expressing the *Cry1Ab* gene were successfully developed through *Agrobacterium*-mediated transformation, which conferred resistance against *M. separata* larvae (Figure 2, Figure 3, Figure 4, Figure 5 and Figure 6). These results suggest the potential for the commercial use of transgenic *Bt* ornamental plants.

Confirming the copy number and integration of transgenes into the plant genome is crucial, as these factors significantly influence gene expression in transgenic plants. The conventional Southern blot is widely used to identify the copy number of integrated transgenes in various plant genomes, including rice [49], cotton [50], cowpea [51], and Torenia [52]. Consistent with these studies, we confirmed the copy number and integration of the *Cry1Ab* gene in the Torenia genome through Southern blot analysis, which revealed a single copy of Cry1Ab DNA insertion in the four Cry1Ab-expressing lines (Figure 3). ELISA is an efficient detection method that has been widely used for the quantitative analysis of Cry proteins in transgenic *Bt* plants [39]. In this study, the levels of *Cry1Ab* transcripts and protein content in the four Cry1Ab-expressing lines were measured using qRT-PCR and ELISA. Our results showed that the *Cry1Ab* transcript levels varied among the four transgenic lines and were positively correlated with the Cry1Ab protein levels in the corresponding transgenic lines, with the highest levels found in Line L1 and the lowest in Line L11 (Figure 4 and Figure 5). The resistance of transgenic *Bt* crops to target pests is typically associated with the levels of insecticidal proteins [53]. Our insect bioassay confirmed that the mortality of *M. separata* larvae fed on transgenic plants correlated mostly with the expression and protein levels of Cry1Ab: line L1, which exhibited the highest expression and protein levels of Cry1Ab, showed the greatest larval mortality, while line L11, with the lowest levels, exhibited the lowest mortality (Figure 6). These results suggest that the *Cry1Ab* gene expressed in the transgenic plants triggers resistance to *M. separata* larvae in Torenia.

Insecticidal toxins derived from *B. thuringiensis* are widely recognized for their high toxicity to the larvae of lepidopteran pests [54,55]. For instance, the commonly used Cry1Ab and Cry1Ac toxins exhibit strong resistance against several insect pests in cotton and maize, including *Helicoverpa armigera*, *Helicoverpa zea*, and *Ostrinia nubilalis* [56,57]. Additionally, Cry1Fa has been shown to be effective in controlling *Spodoptera frugiperda* [58], while Cry1C, Cry1Ab, and Cry1Ac demonstrate high toxicity to the rice pest, *Chilo suppressalis* [59]. Therefore, these highly toxic Bt protein-encoding genes are commercially utilized to develop insect-resistant genetically modified crops targeting lepidopteran pests [54]. For example, maize lines containing single *Bt* genes, such as event TC 1507 with the *Cry1F* gene and event IE09S034 with the *Cry1Ie* gene, exhibit high efficacy in controlling *S. frugiperda* and *M. separata*, respectively [60,61]. In this study, we generated four transgenic Torenia lines expressing the *Cry1Ab* gene, which exhibited high mortality rates of *M. separata* larvae, ranging from 59.9% to 100.0%. Our results are consistent with earlier reports that the *Cry1Ab* gene has been introduced into several crops, resulting in high insecticidal efficacy against *M. separata* [36,60]. However, the effects of these four transgenic lines on insect pests and non-target insects under field conditions were not monitored. Future studies are needed to further investigate the insect resistance of these transgenic plants in urban landscaping environments and their potential impacts on non-target insects, such as natural enemies and honeybees.

Insecticidal Cry proteins produced by *Bt* lead to the formation of reactive oxygen species (ROS), with high levels of ROS causing serious damage to the bodies of the insects [62,63]. To eliminate the overproduced ROS, three key antioxidant enzymes, SOD, POD, and CAT, play a central role in combating oxidative stress induced by Cry proteins in insects [64,65]. Understanding the changes in the activity of these key antioxidant enzymes can provide valuable insights into the underlying mechanisms of insect resistance against Cry proteins. Xie et. al. found that *M. separata* larvae fed a Cry1Ac-containing diet disrupted homeostasis in the protective enzyme activities of SOD, POD, and CAT, resulting in the death of *M. separata* [65]. In our study, the effects of transgenic lines expressing the *Cry1Ab* gene on the activities of SOD, POD, and CAT in *M. separata* were also evaluated. Our results showed that *Cry1Ab* transgenic lines activated the activity of SOD at 12 and 24 h, and CAT at 72 h, while suppressing the activity of SOD at 72 h and POD over time. This is consistent with other reports showing that *Bt* toxins disrupt the dynamic balance of protective enzyme activities, exerting toxicity on Lepidoptera larvae [66,67,68]. Thus, the strong toxicity of Cry1Ab to *M. separata* underscores its potential as a candidate for developing insect-resistant plants, not only for crops, but also for ornamental plants.

In summary, our study demonstrated that *Agrobacterium*-mediated genetic transformation is an effective approach to developing transgenic Torenia plants. Insect bioassays confirmed that *Cry1Ab*-expressing Torenia plants exhibited high resistance to the insect pest, *M. separata*, and influenced the activity of protective enzymes in *M. separata*. Our findings suggest that these four transgenic lines could potentially be utilized for commercial development, offering new insights into the application of *Bt* toxin genes and highlighting their effectiveness in insect pest control for non-food ornamentals. We propose that the genetic development of transgenic ornamental plants expressing toxic protein-encoding genes could be a promising strategy for managing insect pests in non-food ornamental plants.

## 4. Materials and Methods

### 4.1. Vector Construction

The binary vector pBI121 (GenBank accession number AF485783) was used for the overexpression of *Cry1Ab* in the Torenia. The full length of *Cry1Ab* gene was polymerase chain reaction (PCR)-amplified from *B. thuringiensis* strain C3, which was isolated by our lab using specific primers (*Cry1Ab*-F1 and *Cry1Ab*-R1, Table S1) and then ligated into the pBI121 vector under the restriction sites of *Bam*HI and *Sal*I yielding pBI121-*Cry1Ab* (Figure 1A). The *Cry1Ab* gene was driven by the CaMV 35S promoter and NOS terminator. The construct pBI121-*Cry1Ab* also carried a *nptII* gene, which conferred kanamycin resistance as a plant selection marker under the control of a NOS promoter and NOS terminator (Figure 1A). The recombinant vector, pBI121-*Cry1Ab*, was transferred into the *A. tumefaciens* strain LBA4404 by the freeze–thaw method [69] and utilized for the transformation of Torenia leaf segments.

### 4.2. Plant Transformation

Torenia (*Torenia fournieri* L.) cultivar Crown Blue and White was used as the plant material in the present work. In vitro cultures of Torenia were grown in glass bottles containing 30 mL of a half-strength Murashige and Skoog medium (1/2 MS) solidified with 0.8% (*w*/*v*) agar at pH 5.8, under a 16 h light and 8 h dark photoperiod at 25 °C (Figure 2A). The *Agrobacterium*-mediated transformation of Torenia was carried out according to the transformation method [27]. Briefly, leaf segments from 4-week-old in vitro Torenia plants were cut into 0.5 cm× 0.5 cm pieces and infected in the *Agrobacterium* suspension for 10 min at 28 °C. The leaf explants were then properly dried on sterilized filter paper and transferred to a 1/2 MS medium containing 1 mg L^−1^ 6-benzylaminopurine (6-BA) and 100 µg L^−1^ 1-naphthaleneacetic acid (NAA) for co-cultivation in darkness at 28 °C for 7 days. After co-cultivation, the infected explants were washed twice with sterile water and finally in a liquid MS medium containing 200 mg L^−1^ cefotaxime for 15 min to eliminate *Agrobacterium*. Then, they were transferred to the 1/2 MS medium supplemented with 1 mg L^−1^ BA, 100 µg L^−1^ NAA, 200 mg L^−1^ cefotaxime, and 100 mg L^−1^ kanamycin for the selection of transformants. The selection medium was changed every 2 weeks, and after 6 weeks, resistant shoots were transferred to a 1/2 MS medium containing 200 µg L^−1^ IBA, 200 mg L^−1^ cefotaxime, and 100 mg L^−1^ kanamycin for elongation and rooting. The rooted plantlets were individually transplanted into a plastic pot (12 cm in diameter) containing peat moss and maintained at 25 °C under a 16/8 h light/dark cycle with 65% humidity in the greenhouse for further analysis.

### 4.3. Genomic DNA Extraction and PCR Screening

Genomic DNA was extracted from young leaves of kanamycin-resistant and wild-type plants using the Cetyl Trimethyl Ammonium Bromide (CTAB) method [70]. The presence of *Cry1Ab* in the putative transgenic plants was confirmed by PCR amplification [29]. Two specific primers, *Cry1Ab*-F2 and *Cry1Ab*-R2 (Table S1), were designed for the amplification of the 424 bp fragment of *Cry1Ab*. PCR amplification was carried out with a total volume of a 20 µL mixture containing a 1 µL DNA template, 10 µL Premix Taq (TaKaRa Biotechnology (Dalian) Co., Ltd.), 0.5 µL of each of the primers (10 µM), and 8 µL deionized PCR water. The PCR cycle program was as follows: initial denaturation for 3 min at 94 °C, followed by 34 cycles of denaturation at 94 °C for 30 s, annealing at 55 °C, and extension at 72 °C for 1 min, followed by a final 5 min extension at 72 °C. The plasmid pBI121-*Cry1Ab* was used as a positive control. The amplified products were separated by electrophoresis on 1% (*w*/*v*) agarose gel and visualized under ultraviolet light after staining with ethidium bromide.

### 4.4. Southern Blot

Genomic DNA from PCR-positive transgenic lines and wild-type plants was used for Southern blot analysis. Approximately 20 µg of genomic DNA per sample was digested with the *Hin*dIII restriction enzyme at 37 °C overnight, separated on 1% (*w*/*v*) agarose gel at 60 V for 5 h, and then transferred to a positively charged nylon membrane. A 752 bp PCR amplified fragment of *Cry1Ab* (using specific primers Cry1Ab-F3 and Cry1Ab-R3, Table S1) was labeled with DIG-dUTP as a probe with DIG High Prime DNA Labeling and Detection Starter Kit II (Roche, Mannheim, Germany) according to the manufacturer’s instruction. The membrane was hybridized with a DIG-labeled *Cry1Ab* probe following the manufacturer’s protocol.

### 4.5. RNA Extraction and qRT-PCR Analysis

QRT-PCR was performed to determine the expression levels of the *Cry1Ab* gene in transgenic Torenia plants. Total RNA was extracted from young leaves of transgenic and wild-type plants using RNAiso Plus (TaKaRa Biotechnology (Dalian) Co., Ltd.) according to the manufacturer’s protocols. First-strand cDNA was synthesized from 1 µg of total RNA in a 20 µL reaction mixture using the PrimeScript^TM^ RT reagent Kit (Perfect Real Time) (TaKaRa Biotechnology (Dalian) Co., Ltd.) and subsequently used as a template for qRT-PCR with SYBR^®^ *Premix Ex Taq*^TM^ II (Perfect Real Time) (TaKaRa Biotechnology (Dalian) Co., Ltd.) following the manufacturer’s instructions. The specific primers, *Cry1Ab*-qF and *Cry1Ab*-qR, which amplify a 122 bp fragment of the *Cry1Ab* CDS were used for QRT-PCR. *β-Actin*, with specific primers *Actin*-F and *Actin*-R, was used as an internal reference to normalize the expression levels of the target gene as previously reported [34]. Experiments were repeated 3 times. The data were analyzed using the 2^−ΔΔCT^ method as described by Schmittgen and Livak [71]. The primers used for qRT-PCR are listed in Table S2.

### 4.6. Detection of Cry1Ab Protein in Transgenic Plants

The accumulated levels of Cry1Ab protein in the leaves of transgenic and wild-type plants were quantified using an ELISA kit (Sangon Biotech, Cat#E020001, Shanghai, China), following the manufacturer’s protocol. Briefly, 0.05 g of leaf samples was ground in liquid nitrogen and used for protein extraction with the kit’s extraction buffer. The total protein extracts were added into the ELISA plate wells for the Cry1Ab protein detection according to the manufacturer’s instructions. Optical density (OD) values at 450 nm were measured using a Tecan Infinite M200 plate reader (Tecan, Männedorf, Switzerland). The amount of Cry1Ab protein was calculated using a standard curve generated from the Cry1Ab protein standard. The experiment was conducted in six replicates.

### 4.7. Insect Bioassay

The insecticidal activity of the transgenic Torenia lines expressing the *Cry1Ab* toxin gene was evaluated using a no-choice detached leaf feeding bioassay with newly hatched first-instar larvae of the oriental armyworm, *M. separata*. Approximately 800–1000 mg of fresh leaves was placed in plastic Petri dishes lined with moist filter paper, and 10 healthy neonate larvae were carefully released onto the excised leaves using a fine brush. The dishes were sealed with Parafilm to prevent desiccation and maintained in an insect-rearing box at 25 ± 1 °C, with a 12 h photoperiod and 70% relative humidity. Larvae were transferred daily to fresh leaves from intact plants of transgenic lines and wild-type control. The mortality and survival rates were recorded daily until the larvae had either died or pupated. Fifty larvae per transgenic line or wild-type plant were used as a treatment, with each treatment replicated three times.

### 4.8. Determination of Protective Enzyme Activity

The 3rd instar *M. separata* larvae were released onto the leaves of transgenic lines and wild-type plants. Leaf samples were collected at 0, 12, 24, 48, and 72 h post larvae release and then homogenized in phosphate buffer at 4 °C. The homogenates were centrifuged at 4000 g at 4 °C for 10 min, and the supernatants were used for enzyme analysis. The activities of SOD, POD, and CAT were quantified using the detection kit from Nanjing Jiancheng Ltd., Co. (Nanjing, China) according to the manufacturer’s protocol. Each treatment was performed in triplicate and each replicate consisted of three larvae.

### 4.9. Statistical Analysis

Data on the levels of *Cry1Ab* transcripts and protein content in the leaves of four transgenic Torenia lines and wild-type plants, as well as the mortality rate, survival rate, and protective enzyme activity of *M. separata* larvae fed on transgenic lines and wild-type plants, were analyzed using one-way ANOVA followed by Tukey’s HSD post hoc test. All data were statistically analyzed using IBM SPSS Statistics 26 software (IBM Corp., Armonk, NY, USA).

## Data Availability

The original contributions presented in this study are included in the article; further inquiries can be directed to the corresponding author.

## Supplementary Materials

The following supporting information can be downloaded at: https://www.mdpi.com/article/10.3390/plants13243568/s1, Table S1: Specific primers used for cloning and detection of *Cry1Ab* gene. Table S2: Specific primers used for qRT-PCR.
