# Supplementary material for: Genetic Transformation of Torenia fournieri L. with the Bacillus thuringiensis Cry1Ab Gene Confers Resistance to Mythimna separata (Walker)
Source: Plants (Basel). 2024 Dec 20;13(24):3568. doi: 10.3390/plants13243568 (PMC11678925; doi:10.3390/plants13243568)
Supplement: Supplementary file 1 [file plants-13-03568-s001.zip › plants-3338553-supplementary.pdf]

Supplementary Materials:

Genetic Transformation of *Torenia fournieri* L. with the *Bacillus thuringiensis Cry1Ab* Gene Confers Resistance to *Mythimna separata* (Walker)

Lin Chen [1,2], Pei Wang [2,3], Lixia Tan [2], Houhua Li [4] and Dun Wang [2,*]

[1] College of Plant Protection, Yangzhou University, Yangzhou 225009, China; chenlin88@yzu.edu.cn
[2] State Key Laboratory of Crop Stress Biology for Arid Areas, College of Plant Protection, Northwest A&F University, Yangling, Xianyang 712100, China; jlwp0306@163.com (P.W.); tanlixia-000@163.com (L.T.)
[3] Qingdao Smart Village Development Service Center, Qingdao 266000, China
[4] College of Landscape Architecture and Art, Northwest A&F University, Yangling, Xianyang 712100, China; lihouhua@nwafu.edu.cn
* Correspondence: wanghande@nwsuaf.edu.cn; Tel.: +86-029-87091511

Table S1. Specific primers used for cloning and detection of *Cry1Ab* gene

NO.	Name	Sequence (5′-3′)
1	*Cry1Ab*-F1	A*GGATCC*ATGGATAACAATCCGAACATC
2	*Cry1Ab*-R1	GC*AAGCTT*TTATTCCTCCATAAGAAG
3	*Cry1Ab*-F2	GCTCTGGAACTTCTGTCG
4	*Cry1Ab*-R2	CAGCTCATTCACCGCCTT
5	*Cry1Ab*-F3	ACTCTTCCTATATTTACTTTGCCCG
6	*Cry1Ab*-R3	AATGCGTCCCATTGAGAGGG

Table S2. Specific primers used for qRT-PCR

NO.	Name	Sequence (5′-3′)
1	*Cry1Ab*-qF	CCGTTCGGTCCTTGTTGTTCC
2	*Cry1Ab*-qR	CGCAACCTTCTCCATATCCCT
3	*Actin*-F	CTGATTCTCCCTTCCTTAT
4	*Actin*-R	AGCCTCGTCACCAACAT
